# (4′-Chloro-2,2′:6′,2′′-terpyridine-κ^3^ *N*,*N*′,*N*′′)bis(nitrato-κ*O*)zinc(II)

**DOI:** 10.1107/S2414314620013449

**Published:** 2020-10-13

**Authors:** Rafael A. Adrian, Daniela Canales, Hadi D. Arman

**Affiliations:** a Department of Chemistry and Biochemistry, University of the Incarnate Word, San Antonio, TX 78209, USA; b Department of Chemistry, The University of Texas at San Antonio, San Antonio, TX 78249, USA; Katholieke Universiteit Leuven, Belgium

**Keywords:** crystal structure, zinc atom, nitrate ions, 4-chloro­terpyridine

## Abstract

In the title complex, [Zn(C_15_H_10_ClN_3_)(NO_3_)_2_], the central zinc(II) atom is penta­coordinated by two O-bonded nitrate ions and three N atoms of the 4′-chloro-2,2′:6′,2′′-terpyridine ligand in a slightly distorted trigonal–bipyramidal shape. The main feature of the title complex packing is the presence of offset *π*–*π* inter­actions between the terpyridine rings in different layers. This structure constitutes the first known example of a zinc(II) 4-chloro­terpyridine complex with coordinating nitrate ions.

## Abstract 2

The coordination of the Zn^II^ central atom in the title complex, [Zn(C_15_H_10_ClN_3_)(NO_3_)_2_], exists in a distorted trigonal bipyramidal geometry defined by the nitro­gen atoms of the 4-chloro­terpyridine ligand and two monodentate nitrate groups.

## Graphical abstract

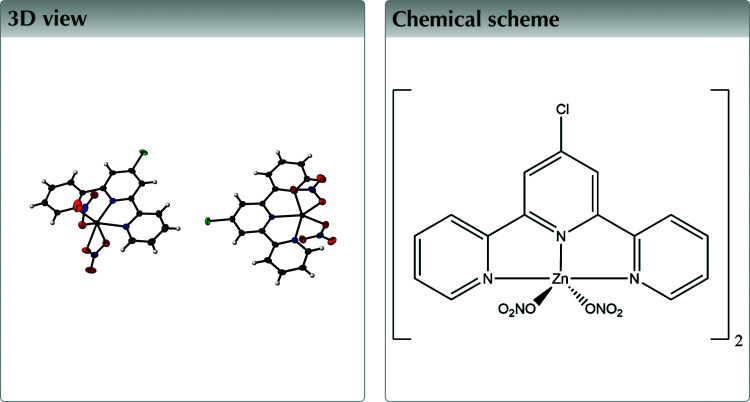

## Structure description

Metal–organic complexes of 4′-chloro-2,2′:6′,2′′-terpyridine have been investigated because of their ability to generate supra­molecular frameworks (Huang & Qian, 2008 ▸), and for their photosensitivity properties (Dutta *et al.*, 2019 ▸). As part of our research related to the coordination chemistry of metal ions with bi­pyridine and terpyridine ligands, hereto we report the synthesis and structure of the title zinc(II) complex.

**Figure 1:**
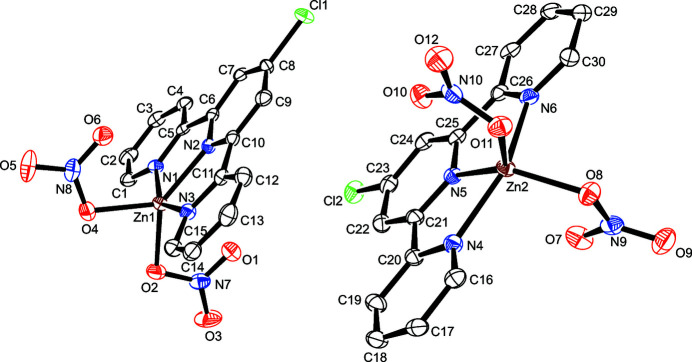
The mol­ecular structure of the title compound with displacement ellipsoids drawn at the 50% probability level; H atoms are omitted for clarity.

The asymmetric unit of the title compound, [Zn(C_15_H_10_ClN_3_)(NO_3_)_2_], contains two nearly identical complexes with an r.m.s. deviation for overlay of 0.1679 Å (calculated using *Mercury*; Macrae *et al.*, 2020 ▸), with a five-coordinate distorted trigonal–bipyramidal environment around the zinc(II) atom. The central zinc(II) atom is chelated by the nitro­gen atoms of the 4′-chloro-2,2′:6′,2′′-terpyridine ligand and additionally coordinated by two O-bonded nitrate ions (Fig. 1 ▸). The coordinated oxygen atoms of the nitrate ions and the central nitro­gen atom of the terpyridine ligand lie in the equatorial plane, while the other two nitro­gen atoms of the terpyridine ligand are in the axial positions with longer Zn—N bond lengths. All relevant bonds and angles are presented in Table 1 ▸.

**Figure 2:**
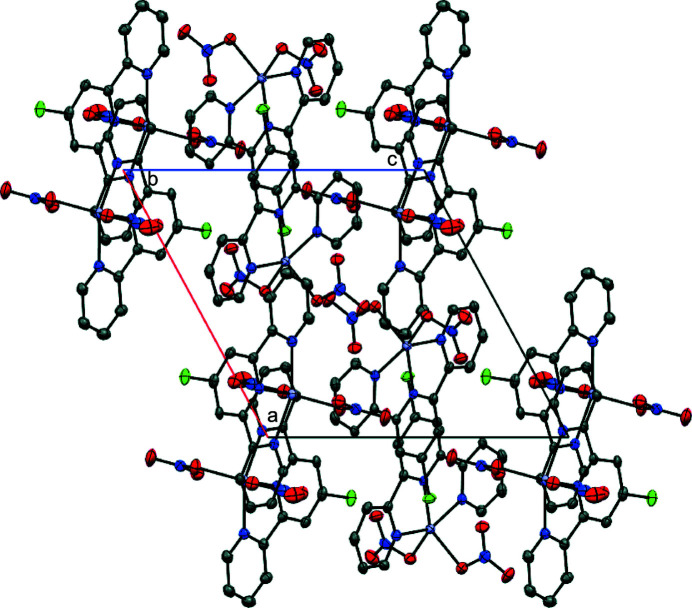
Perspective view of the packing structure of the title complex along the crystallographic *b* axis; H atoms are omitted for clarity.

**Figure 3:**
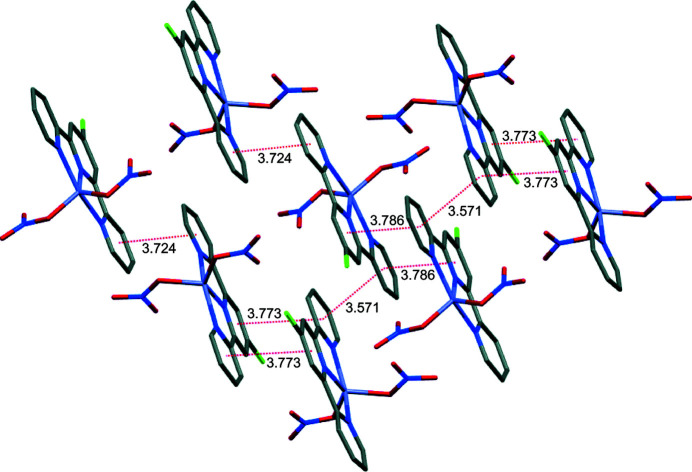
Capped sticks representation of the title mol­ecule showing the *Cg*⋯*Cg* distances between pyridine rings; H atoms are omitted for clarity

The title complex packs into layers in the *ac* plane that are aligned along the *b*-axis direction (Fig. 2 ▸). Contiguous pyridine rings show π–π stacking inter­actions, with centroid-to-centroid (*Cg*⋯*Cg*) distances ranging from 3.571 (1) to 3.786 (1) Å as shown in Fig. 3 ▸, and offset distances ranging from 1.073 to 1.637 Å. The *Cg⋯*Cg** distance is influenced by the relative positioning of the chlorine atom of the terpyridine unit.

## Synthesis and crystallization

Solid 4′-chloro-2,2′:6′,2′′-terpyridine (0.100 g, 0.374 mmol) was added to ZnCl_2_ (0.051 g, 0.37 mmol) in 50.0 ml of methanol and the resulting solution was stirred without heating for 2 h. AgNO_3_ (0.127 g, 0.748 mmol) was added to the clear solution and stirred without heating for 45 minutes. After the removal of AgCl by filtration using a 0.45 µm PTFE syringe filter, the resulting clear solution was rotovaped to dryness. The dried product was then redissolved in 10.0 ml of aceto­nitrile and the clear solution was used to grow crystals by vapor diffusion with diethyl ether at 278 K.

## Refinement

Crystal data, data collection and structure refinement details are summarized in Table 2 ▸.

## Supplementary Material

Crystal structure: contains datablock(s) general, I. DOI: 10.1107/S2414314620013449/vm4047sup1.cif

Structure factors: contains datablock(s) I. DOI: 10.1107/S2414314620013449/vm4047Isup2.hkl

Supporting information file. DOI: 10.1107/S2414314620013449/vm4047Isup3.mol

3D View. DOI: 10.1107/S2414314620013449/vm4047sup4.ps

CCDC reference: 2035884

Additional supporting information:  crystallographic information; 3D view; checkCIF report

## Figures and Tables

**Table 1:** Selected geometric parameters (Å, °)

Zn1—O2	2.064 (2)	Zn2—O8	2.043 (2)
Zn1—O4	2.048 (2)	Zn2—O11	2.049 (2)
Zn1—N1	2.136 (2)	Zn2—N4	2.138 (2)
Zn1—N2	2.083 (2)	Zn2—N5	2.068 (2)
Zn1—N3	2.139 (3)	Zn2—N6	2.133 (2)
			
O4—Zn1—O2	86.69 (9)	O8—Zn2—O11	86.69 (9)
O4—Zn1—N2	132.17 (9)	O8—Zn2—N5	137.93 (9)
O2—Zn1—N2	140.99 (9)	O11—Zn2—N5	135.36 (9)
O4—Zn1—N1	100.03 (9)	O8—Zn2—N6	98.03 (9)
O2—Zn1—N1	96.13 (10)	O11—Zn2—N6	100.19 (9)
N2—Zn1—N1	76.20 (9)	N5—Zn2—N6	76.56 (10)
O4—Zn1—N3	97.01 (9)	O8—Zn2—N4	103.33 (9)
O2—Zn1—N3	106.86 (10)	O11—Zn2—N4	98.17 (9)
N2—Zn1—N3	75.94 (9)	N5—Zn2—N4	75.97 (9)
N1—Zn1—N3	152.05 (10)	N6—Zn2—N4	152.49 (10)

**Table 2:** Experimental details

Crystal data	
Chemical formula	[Zn(C_15_H_10_ClN_3_)(NO_3_)_2_]
*M*_r_	457.10
Crystal system, space group	Triclinic, *P* 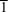
Temperature (K)	98
*a*, *b*, *c* (Å)	12.1001 (19), 13.3139 (18), 13.4869 (12)
α, β, γ (°)	62.471 (9), 63.694 (7), 87.388 (11)
*V* (Å^3^)	1692.2 (4)
*Z*	4
Radiation type	Mo *K*α
μ (mm^−1^)	1.66
Crystal size (mm)	0.35 × 0.20 × 0.05

Data collection	
Diffractometer	Rigaku Saturn724
Absorption correction	Multi-scan (*ABSCOR*; Higashi, 1995 ▸)
*T*_min_, *T*_max_	0.780, 1.000
No. of measured, independent and observed [*I* > 2σ(*I*)] reflections	12078, 6600, 5823
*R*_int_	0.080
(sin θ/λ)_max_ (Å^−1^)	0.617

Refinement	
*R*[*F* ^2^ > 2σ(*F* ^2^)], *wR*(*F* ^2^), *S*	0.043, 0.109, 1.01
No. of reflections	6600
No. of parameters	505
H-atom treatment	H-atom parameters constrained
Δρ_max_, Δρ_min_ (e Å^−3^)	0.75, −0.91

Computer programs: *CrystalClear-SM Expert* (Rigaku, 2010 ▸), *SHELXS97* and *SHELXL97* (Sheldrick, 2008 ▸), *ORTEPII* (Johnson, 1976 ▸), *Mercury* (Macrae *et al.*, 2020 ▸), *DIAMOND* (Brandenburg, 2020 ▸) and *publCIF* (Westrip, 2010 ▸).

## full crystallographic data

### Crystal data

**Table:**

[Zn(NO_3_)_2_(C_15_H_10_ClN_3_)]	*Z* = 4
*M**_r_* = 457.10	*F*(000) = 920
Triclinic, *P*1	*D*_x_ = 1.794 Mg m^−^^3^
Hall symbol: -P 1	Mo *K*α radiation, λ = 0.71073 Å
*a* = 12.1001 (19) Å	Cell parameters from 6364 reflections
*b* = 13.3139 (18) Å	θ = 2.9–40.1°
*c* = 13.4869 (12) Å	µ = 1.66 mm^−^^1^
α = 62.471 (9)°	*T* = 98 K
β = 63.694 (7)°	Platelet, colourless
γ = 87.388 (11)°	0.35 × 0.20 × 0.05 mm
*V* = 1692.2 (4) Å^3^	

### Data collection

**Table:**

Rigaku Saturn724 diffractometer	6600 independent reflections
Radiation source: sealed tube	5823 reflections with *I* > 2σ(*I*)
Graphite monochromator	*R*_int_ = 0.080
Detector resolution: 28.5714 pixels mm^-1^	θ_max_ = 26.0°, θ_min_ = 2.9°
ω scans	*h* = −14→14
Absorption correction: multi-scan (ABSCOR; Higashi, 1995)	*k* = −16→16
*T*_min_ = 0.780, *T*_max_ = 1.000	*l* = −16→15
12078 measured reflections	

### Refinement

**Table:**

Refinement on *F*^2^	Primary atom site location: structure-invariant direct methods
Least-squares matrix: full	Secondary atom site location: difference Fourier map
*R*[*F*^2^ > 2σ(*F*^2^)] = 0.043	Hydrogen site location: inferred from neighbouring sites
*wR*(*F*^2^) = 0.109	H-atom parameters constrained
*S* = 1.01	*w* = 1/[σ^2^(*F*_o_^2^) + (0.050*P*)^2^ + 1.5*P*] where *P* = (*F*_o_^2^ + 2*F*_c_^2^)/3
6600 reflections	(Δ/σ)_max_ = 0.001
505 parameters	Δρ_max_ = 0.75 e Å^−^^3^
0 restraints	Δρ_min_ = −0.91 e Å^−^^3^

### Special details

**Table:**

Geometry. All esds (except the esd in the dihedral angle between two l.s. planes) are estimated using the full covariance matrix. The cell esds are taken into account individually in the estimation of esds in distances, angles and torsion angles; correlations between esds in cell parameters are only used when they are defined by crystal symmetry. An approximate (isotropic) treatment of cell esds is used for estimating esds involving l.s. planes.
Refinement. Refinement of F^2^ against ALL reflections. The weighted R-factor wR and goodness of fit S are based on F^2^, conventional R-factors R are based on F, with F set to zero for negative F^2^. The threshold expression of F^2^ > 2sigma(F^2^) is used only for calculating R-factors(gt) etc. and is not relevant to the choice of reflections for refinement. R-factors based on F^2^ are statistically about twice as large as those based on F, and R- factors based on ALL data will be even larger. All C-bound H-atoms were placed on calculated positions and were refined with *U*_iso_(H) = 1.2*U*_eq_(C).

### Fractional atomic coordinates and isotropic or equivalent isotropic displacement parameters (Å^2^)

**Table:**

	*x*	*y*	*z*	*U*_iso_*/*U*_eq_	
Zn2	0.34214 (3)	0.94668 (3)	0.37421 (3)	0.01727 (10)	
Zn1	−0.83871 (3)	0.31255 (3)	0.84356 (3)	0.01784 (10)	
Cl2	−0.22788 (7)	0.71144 (7)	0.57358 (7)	0.02664 (18)	
Cl1	−0.77312 (8)	0.52743 (7)	1.16640 (7)	0.02960 (19)	
C20	0.2606 (3)	0.8239 (2)	0.2729 (3)	0.0182 (6)	
N2	−0.8173 (2)	0.3886 (2)	0.9403 (2)	0.0169 (5)	
N5	0.1619 (2)	0.8686 (2)	0.4390 (2)	0.0163 (5)	
N1	−1.0252 (2)	0.2792 (2)	0.9920 (2)	0.0190 (5)	
N6	0.2305 (2)	0.9699 (2)	0.5337 (2)	0.0188 (5)	
C10	−0.7033 (3)	0.4357 (2)	0.9060 (3)	0.0181 (6)	
C25	0.0667 (3)	0.8670 (2)	0.5419 (3)	0.0172 (6)	
C24	−0.0557 (3)	0.8156 (2)	0.5885 (3)	0.0199 (6)	
H24A	−0.1220	0.8139	0.6598	0.024*	
O11	0.4890 (2)	0.89317 (19)	0.4088 (2)	0.0253 (5)	
C21	0.1425 (3)	0.8170 (2)	0.3820 (3)	0.0184 (6)	
C5	−1.0385 (3)	0.3216 (2)	1.0698 (3)	0.0180 (6)	
N3	−0.6406 (2)	0.3777 (2)	0.7475 (2)	0.0199 (5)	
C26	0.1059 (3)	0.9247 (2)	0.5963 (3)	0.0184 (6)	
C11	−0.6007 (3)	0.4316 (2)	0.7931 (3)	0.0187 (6)	
O8	0.4539 (2)	1.10805 (18)	0.2536 (2)	0.0257 (5)	
N4	0.3661 (2)	0.8809 (2)	0.2498 (2)	0.0194 (5)	
C9	−0.6837 (3)	0.4814 (3)	0.9727 (3)	0.0214 (6)	
H9A	−0.6040	0.5161	0.9475	0.026*	
C27	0.0229 (3)	0.9340 (3)	0.7018 (3)	0.0204 (6)	
H27A	−0.0627	0.9038	0.7432	0.024*	
N10	0.4460 (2)	0.7897 (2)	0.5056 (2)	0.0230 (6)	
C4	−1.1562 (3)	0.3093 (3)	1.1677 (3)	0.0212 (6)	
H4A	−1.1641	0.3391	1.2203	0.025*	
C22	0.0232 (3)	0.7647 (2)	0.4214 (3)	0.0194 (6)	
H22A	0.0098	0.7298	0.3807	0.023*	
O10	0.3348 (2)	0.74424 (19)	0.5505 (2)	0.0271 (5)	
C30	0.2751 (3)	1.0227 (3)	0.5761 (3)	0.0214 (6)	
H30A	0.3609	1.0525	0.5335	0.026*	
C6	−0.9194 (3)	0.3797 (2)	1.0434 (3)	0.0173 (6)	
C1	−1.1267 (3)	0.2224 (3)	1.0112 (3)	0.0219 (6)	
H1A	−1.1169	0.1922	0.9585	0.026*	
C23	−0.0756 (3)	0.7669 (3)	0.5251 (3)	0.0206 (6)	
N9	0.3979 (2)	1.1808 (2)	0.1981 (2)	0.0239 (6)	
C16	0.4772 (3)	0.8890 (3)	0.1554 (3)	0.0211 (6)	
H16A	0.5497	0.9285	0.1397	0.025*	
C7	−0.9085 (3)	0.4211 (2)	1.1173 (3)	0.0192 (6)	
H7A	−0.9782	0.4144	1.1894	0.023*	
O1	−0.8653 (2)	0.5083 (2)	0.7003 (2)	0.0298 (5)	
C3	−1.2611 (3)	0.2515 (3)	1.1844 (3)	0.0245 (7)	
H3A	−1.3409	0.2432	1.2478	0.029*	
O4	−0.8284 (2)	0.15038 (19)	0.8647 (2)	0.0273 (5)	
C29	0.1981 (3)	1.0345 (3)	0.6812 (3)	0.0239 (7)	
H29A	0.2313	1.0718	0.7087	0.029*	
O6	−0.7917 (2)	0.1417 (2)	1.0121 (2)	0.0300 (5)	
N8	−0.8006 (2)	0.0932 (2)	0.9554 (3)	0.0270 (6)	
N7	−0.8941 (2)	0.4541 (2)	0.6577 (2)	0.0260 (6)	
C19	0.2642 (3)	0.7731 (3)	0.2021 (3)	0.0226 (6)	
H19A	0.1906	0.7336	0.2197	0.027*	
C8	−0.7891 (3)	0.4726 (2)	1.0785 (3)	0.0207 (6)	
O2	−0.8891 (2)	0.3455 (2)	0.7054 (2)	0.0307 (5)	
C15	−0.5539 (3)	0.3659 (3)	0.6498 (3)	0.0241 (7)	
H15A	−0.5808	0.3289	0.6181	0.029*	
O7	0.2949 (2)	1.1410 (2)	0.2176 (2)	0.0315 (5)	
O12	0.5158 (2)	0.7405 (2)	0.5489 (2)	0.0339 (6)	
C2	−1.2463 (3)	0.2063 (3)	1.1062 (3)	0.0258 (7)	
H2A	−1.3154	0.1660	1.1174	0.031*	
C12	−0.4751 (3)	0.4759 (3)	0.7412 (3)	0.0239 (6)	
H12A	−0.4500	0.5143	0.7727	0.029*	
C17	0.4879 (3)	0.8407 (3)	0.0806 (3)	0.0217 (6)	
H17A	0.5660	0.8475	0.0160	0.026*	
C18	0.3794 (3)	0.7822 (3)	0.1045 (3)	0.0243 (7)	
H18A	0.3837	0.7492	0.0557	0.029*	
O9	0.4494 (2)	1.2831 (2)	0.1288 (2)	0.0354 (6)	
C28	0.0709 (3)	0.9895 (3)	0.7440 (3)	0.0242 (7)	
H28A	0.0172	0.9963	0.8146	0.029*	
C14	−0.4266 (3)	0.4064 (3)	0.5947 (3)	0.0254 (7)	
H14A	−0.3691	0.3963	0.5278	0.030*	
O5	−0.7842 (3)	−0.0062 (2)	0.9827 (3)	0.0453 (7)	
O3	−0.9241 (3)	0.4983 (3)	0.5740 (2)	0.0442 (7)	
C13	−0.3865 (3)	0.4620 (3)	0.6407 (3)	0.0275 (7)	
H13A	−0.3012	0.4901	0.6052	0.033*	

### Atomic displacement parameters (Å^2^)

**Table:**

	*U*^11^	*U*^22^	*U*^33^	*U*^12^	*U*^13^	*U*^23^
Zn2	0.01517 (18)	0.02157 (19)	0.01686 (18)	0.00285 (14)	−0.00808 (14)	−0.01030 (15)
Zn1	0.01927 (19)	0.02017 (18)	0.01752 (18)	0.00398 (14)	−0.00944 (14)	−0.01119 (15)
Cl2	0.0179 (4)	0.0258 (4)	0.0308 (4)	−0.0001 (3)	−0.0117 (3)	−0.0096 (3)
Cl1	0.0434 (5)	0.0315 (4)	0.0277 (4)	0.0076 (4)	−0.0234 (4)	−0.0186 (3)
C20	0.0222 (15)	0.0180 (14)	0.0198 (14)	0.0070 (11)	−0.0142 (12)	−0.0096 (12)
N2	0.0196 (12)	0.0144 (11)	0.0179 (12)	0.0063 (9)	−0.0100 (10)	−0.0081 (10)
N5	0.0159 (12)	0.0170 (11)	0.0166 (11)	0.0047 (9)	−0.0098 (10)	−0.0070 (10)
N1	0.0219 (13)	0.0194 (12)	0.0178 (12)	0.0046 (10)	−0.0120 (10)	−0.0084 (10)
N6	0.0226 (13)	0.0190 (12)	0.0158 (11)	0.0064 (10)	−0.0110 (10)	−0.0077 (10)
C10	0.0205 (14)	0.0139 (13)	0.0200 (14)	0.0052 (11)	−0.0115 (12)	−0.0068 (11)
C25	0.0175 (14)	0.0154 (13)	0.0143 (13)	0.0036 (11)	−0.0080 (11)	−0.0036 (11)
C24	0.0186 (14)	0.0184 (14)	0.0167 (14)	0.0055 (11)	−0.0084 (12)	−0.0044 (12)
O11	0.0231 (11)	0.0289 (12)	0.0243 (11)	0.0055 (9)	−0.0136 (9)	−0.0112 (10)
C21	0.0231 (15)	0.0178 (14)	0.0179 (14)	0.0069 (12)	−0.0126 (12)	−0.0089 (12)
C5	0.0221 (15)	0.0146 (13)	0.0152 (13)	0.0065 (11)	−0.0119 (12)	−0.0031 (11)
N3	0.0212 (13)	0.0189 (12)	0.0194 (12)	0.0048 (10)	−0.0101 (11)	−0.0090 (10)
C26	0.0238 (15)	0.0154 (13)	0.0163 (13)	0.0064 (11)	−0.0117 (12)	−0.0062 (11)
C11	0.0218 (15)	0.0142 (13)	0.0191 (14)	0.0047 (11)	−0.0107 (12)	−0.0066 (11)
O8	0.0222 (11)	0.0234 (11)	0.0257 (11)	0.0031 (9)	−0.0114 (9)	−0.0079 (10)
N4	0.0207 (13)	0.0190 (12)	0.0192 (12)	0.0052 (10)	−0.0102 (10)	−0.0095 (10)
C9	0.0241 (16)	0.0191 (14)	0.0226 (15)	0.0047 (12)	−0.0154 (13)	−0.0074 (12)
C27	0.0215 (15)	0.0191 (14)	0.0139 (13)	0.0063 (12)	−0.0084 (12)	−0.0035 (12)
N10	0.0274 (14)	0.0260 (13)	0.0266 (13)	0.0091 (11)	−0.0159 (12)	−0.0185 (12)
C4	0.0237 (15)	0.0197 (14)	0.0169 (14)	0.0073 (12)	−0.0096 (12)	−0.0068 (12)
C22	0.0209 (15)	0.0185 (14)	0.0229 (15)	0.0053 (12)	−0.0141 (12)	−0.0100 (12)
O10	0.0249 (12)	0.0285 (12)	0.0315 (12)	0.0039 (9)	−0.0136 (10)	−0.0172 (10)
C30	0.0236 (15)	0.0209 (14)	0.0215 (14)	0.0037 (12)	−0.0128 (13)	−0.0098 (12)
C6	0.0236 (15)	0.0122 (13)	0.0157 (13)	0.0066 (11)	−0.0107 (12)	−0.0057 (11)
C1	0.0239 (15)	0.0224 (15)	0.0186 (14)	0.0009 (12)	−0.0124 (13)	−0.0071 (12)
C23	0.0185 (14)	0.0179 (14)	0.0223 (14)	0.0024 (11)	−0.0128 (12)	−0.0047 (12)
N9	0.0217 (13)	0.0293 (14)	0.0175 (12)	0.0061 (11)	−0.0047 (11)	−0.0136 (11)
C16	0.0194 (15)	0.0214 (14)	0.0181 (14)	0.0046 (12)	−0.0075 (12)	−0.0081 (12)
C7	0.0269 (16)	0.0153 (13)	0.0145 (13)	0.0068 (12)	−0.0120 (12)	−0.0050 (11)
O1	0.0297 (12)	0.0323 (12)	0.0317 (12)	0.0055 (10)	−0.0147 (10)	−0.0189 (11)
C3	0.0217 (15)	0.0244 (15)	0.0184 (14)	0.0074 (13)	−0.0084 (12)	−0.0054 (13)
O4	0.0328 (12)	0.0232 (11)	0.0311 (12)	0.0076 (9)	−0.0161 (10)	−0.0165 (10)
C29	0.0349 (18)	0.0206 (15)	0.0195 (14)	0.0061 (13)	−0.0155 (14)	−0.0100 (12)
O6	0.0328 (13)	0.0292 (12)	0.0293 (12)	0.0056 (10)	−0.0173 (11)	−0.0130 (10)
N8	0.0219 (14)	0.0189 (13)	0.0336 (15)	0.0045 (11)	−0.0105 (12)	−0.0106 (12)
N7	0.0230 (14)	0.0336 (15)	0.0203 (13)	0.0090 (12)	−0.0101 (11)	−0.0129 (12)
C19	0.0273 (16)	0.0255 (15)	0.0248 (15)	0.0090 (13)	−0.0174 (13)	−0.0151 (13)
C8	0.0340 (17)	0.0153 (13)	0.0226 (15)	0.0084 (12)	−0.0191 (13)	−0.0114 (12)
O2	0.0444 (14)	0.0314 (12)	0.0274 (12)	0.0133 (11)	−0.0234 (11)	−0.0172 (10)
C15	0.0277 (16)	0.0234 (15)	0.0216 (15)	0.0065 (13)	−0.0111 (13)	−0.0122 (13)
O7	0.0220 (12)	0.0457 (14)	0.0354 (13)	0.0094 (11)	−0.0143 (10)	−0.0261 (12)
O12	0.0384 (14)	0.0349 (13)	0.0426 (14)	0.0193 (11)	−0.0310 (12)	−0.0195 (12)
C2	0.0209 (15)	0.0262 (16)	0.0239 (15)	0.0021 (13)	−0.0141 (13)	−0.0042 (13)
C12	0.0229 (16)	0.0217 (15)	0.0277 (16)	0.0037 (12)	−0.0132 (13)	−0.0114 (13)
C17	0.0254 (16)	0.0238 (15)	0.0163 (13)	0.0091 (12)	−0.0104 (12)	−0.0102 (12)
C18	0.0338 (17)	0.0254 (15)	0.0207 (15)	0.0095 (13)	−0.0158 (14)	−0.0142 (13)
O9	0.0394 (14)	0.0234 (12)	0.0286 (12)	0.0058 (11)	−0.0106 (11)	−0.0074 (10)
C28	0.0329 (17)	0.0229 (15)	0.0146 (14)	0.0084 (13)	−0.0101 (13)	−0.0091 (12)
C14	0.0278 (17)	0.0212 (15)	0.0209 (15)	0.0065 (13)	−0.0086 (13)	−0.0089 (13)
O5	0.0421 (15)	0.0185 (12)	0.0657 (18)	0.0089 (11)	−0.0240 (14)	−0.0155 (12)
O3	0.0540 (17)	0.0570 (17)	0.0340 (14)	0.0267 (14)	−0.0333 (13)	−0.0213 (13)
C13	0.0224 (16)	0.0219 (15)	0.0296 (17)	0.0052 (13)	−0.0094 (14)	−0.0093 (14)

### Geometric parameters (Å, º)

**Table:**

Zn1—O2	2.064 (2)	C27—H27A	0.9300
Zn1—O4	2.048 (2)	N10—O12	1.230 (3)
Zn1—N1	2.136 (2)	N10—O10	1.248 (3)
Zn1—N2	2.083 (2)	C4—C3	1.390 (5)
Zn1—N3	2.139 (3)	C4—H4A	0.9300
Zn2—O8	2.043 (2)	C22—C23	1.393 (4)
Zn2—O11	2.049 (2)	C22—H22A	0.9300
Zn2—N4	2.138 (2)	C30—C29	1.387 (4)
Zn2—N5	2.068 (2)	C30—H30A	0.9300
Zn2—N6	2.133 (2)	C6—C7	1.394 (4)
Cl2—C23	1.725 (3)	C1—C2	1.383 (4)
Cl1—C8	1.730 (3)	C1—H1A	0.9300
C20—N4	1.349 (4)	N9—O9	1.226 (3)
C20—C19	1.389 (4)	N9—O7	1.242 (4)
C20—C21	1.497 (4)	C16—C17	1.386 (4)
N2—C10	1.325 (4)	C16—H16A	0.9300
N2—C6	1.347 (4)	C7—C8	1.385 (4)
N5—C21	1.335 (4)	C7—H7A	0.9300
N5—C25	1.347 (4)	O1—N7	1.248 (4)
N1—C1	1.330 (4)	C3—C2	1.384 (5)
N1—C5	1.355 (4)	C3—H3A	0.9300
N6—C30	1.338 (4)	O4—N8	1.291 (4)
N6—C26	1.355 (4)	C29—C28	1.380 (5)
C10—C9	1.395 (4)	C29—H29A	0.9300
C10—C11	1.502 (4)	O6—N8	1.245 (4)
C25—C24	1.388 (4)	N8—O5	1.232 (3)
C25—C26	1.487 (4)	N7—O3	1.217 (4)
C24—C23	1.385 (4)	N7—O2	1.294 (4)
C24—H24A	0.9300	C19—C18	1.390 (4)
O11—N10	1.294 (3)	C19—H19A	0.9300
C21—C22	1.386 (4)	C15—C14	1.380 (5)
C5—C4	1.398 (4)	C15—H15A	0.9300
C5—C6	1.482 (4)	C2—H2A	0.9300
N3—C15	1.346 (4)	C12—C13	1.395 (4)
N3—C11	1.354 (4)	C12—H12A	0.9300
C26—C27	1.391 (4)	C17—C18	1.386 (5)
C11—C12	1.380 (4)	C17—H17A	0.9300
O8—N9	1.295 (3)	C18—H18A	0.9300
N4—C16	1.345 (4)	C28—H28A	0.9300
C9—C8	1.385 (4)	C14—C13	1.376 (5)
C9—H9A	0.9300	C14—H14A	0.9300
C27—C28	1.387 (5)	C13—H13A	0.9300
			
O4—Zn1—O2	86.69 (9)	O10—N10—O11	117.9 (3)
O4—Zn1—N2	132.17 (9)	C3—C4—C5	118.2 (3)
O2—Zn1—N2	140.99 (9)	C3—C4—H4A	120.9
O4—Zn1—N1	100.03 (9)	C5—C4—H4A	120.9
O2—Zn1—N1	96.13 (10)	C21—C22—C23	116.6 (3)
N2—Zn1—N1	76.20 (9)	C21—C22—H22A	121.7
O4—Zn1—N3	97.01 (9)	C23—C22—H22A	121.7
O2—Zn1—N3	106.86 (10)	N6—C30—C29	122.5 (3)
N2—Zn1—N3	75.94 (9)	N6—C30—H30A	118.8
N1—Zn1—N3	152.05 (10)	C29—C30—H30A	118.8
O8—Zn2—O11	86.69 (9)	N2—C6—C7	120.8 (3)
O8—Zn2—N5	137.93 (9)	N2—C6—C5	114.2 (2)
O11—Zn2—N5	135.36 (9)	C7—C6—C5	125.0 (3)
O8—Zn2—N6	98.03 (9)	N1—C1—C2	122.8 (3)
O11—Zn2—N6	100.19 (9)	N1—C1—H1A	118.6
N5—Zn2—N6	76.56 (10)	C2—C1—H1A	118.6
O8—Zn2—N4	103.33 (9)	C24—C23—C22	121.9 (3)
O11—Zn2—N4	98.17 (9)	C24—C23—Cl2	118.8 (2)
N5—Zn2—N4	75.97 (9)	C22—C23—Cl2	119.3 (2)
N6—Zn2—N4	152.49 (10)	O9—N9—O7	124.1 (3)
N4—C20—C19	121.6 (3)	O9—N9—O8	118.8 (3)
N4—C20—C21	114.8 (2)	O7—N9—O8	117.1 (3)
C19—C20—C21	123.5 (3)	N4—C16—C17	122.6 (3)
C10—N2—C6	121.5 (3)	N4—C16—H16A	118.7
C10—N2—Zn1	119.70 (19)	C17—C16—H16A	118.7
C6—N2—Zn1	118.6 (2)	C8—C7—C6	117.0 (3)
C21—N5—C25	121.2 (3)	C8—C7—H7A	121.5
C21—N5—Zn2	119.96 (19)	C6—C7—H7A	121.5
C25—N5—Zn2	118.8 (2)	C2—C3—C4	119.6 (3)
C1—N1—C5	118.9 (3)	C2—C3—H3A	120.2
C1—N1—Zn1	125.0 (2)	C4—C3—H3A	120.2
C5—N1—Zn1	116.10 (19)	N8—O4—Zn1	107.28 (18)
C30—N6—C26	119.1 (2)	C28—C29—C30	118.3 (3)
C30—N6—Zn2	125.0 (2)	C28—C29—H29A	120.9
C26—N6—Zn2	115.86 (19)	C30—C29—H29A	120.9
N2—C10—C9	121.7 (3)	O5—N8—O6	122.5 (3)
N2—C10—C11	113.7 (3)	O5—N8—O4	119.5 (3)
C9—C10—C11	124.6 (3)	O6—N8—O4	118.0 (2)
N5—C25—C24	120.6 (3)	O3—N7—O1	123.5 (3)
N5—C25—C26	114.1 (3)	O3—N7—O2	119.7 (3)
C24—C25—C26	125.3 (3)	O1—N7—O2	116.7 (3)
C23—C24—C25	117.6 (3)	C20—C19—C18	119.0 (3)
C23—C24—H24A	121.2	C20—C19—H19A	120.5
C25—C24—H24A	121.2	C18—C19—H19A	120.5
N10—O11—Zn2	106.77 (18)	C9—C8—C7	122.4 (3)
N5—C21—C22	121.9 (3)	C9—C8—Cl1	119.6 (2)
N5—C21—C20	113.2 (3)	C7—C8—Cl1	118.0 (2)
C22—C21—C20	124.9 (3)	N7—O2—Zn1	104.76 (18)
N1—C5—C4	121.8 (3)	N3—C15—C14	122.7 (3)
N1—C5—C6	114.8 (2)	N3—C15—H15A	118.6
C4—C5—C6	123.4 (3)	C14—C15—H15A	118.6
C15—N3—C11	118.3 (3)	C1—C2—C3	118.7 (3)
C15—N3—Zn1	125.6 (2)	C1—C2—H2A	120.7
C11—N3—Zn1	116.06 (19)	C3—C2—H2A	120.7
N6—C26—C27	121.5 (3)	C11—C12—C13	118.9 (3)
N6—C26—C25	114.7 (2)	C11—C12—H12A	120.5
C27—C26—C25	123.8 (3)	C13—C12—H12A	120.5
N3—C11—C12	121.9 (3)	C18—C17—C16	118.3 (3)
N3—C11—C10	114.6 (3)	C18—C17—H17A	120.8
C12—C11—C10	123.5 (3)	C16—C17—H17A	120.8
N9—O8—Zn2	110.87 (18)	C17—C18—C19	119.5 (3)
C16—N4—C20	119.0 (3)	C17—C18—H18A	120.2
C16—N4—Zn2	125.0 (2)	C19—C18—H18A	120.2
C20—N4—Zn2	116.02 (19)	C29—C28—C27	120.1 (3)
C8—C9—C10	116.6 (3)	C29—C28—H28A	119.9
C8—C9—H9A	121.7	C27—C28—H28A	119.9
C10—C9—H9A	121.7	C13—C14—C15	118.8 (3)
C28—C27—C26	118.5 (3)	C13—C14—H14A	120.6
C28—C27—H27A	120.8	C15—C14—H14A	120.6
C26—C27—H27A	120.8	C14—C13—C12	119.3 (3)
O12—N10—O10	123.0 (3)	C14—C13—H13A	120.4
O12—N10—O11	119.1 (3)	C12—C13—H13A	120.4
